# Evidence for integrating eye health into primary health care in Africa: a health systems strengthening approach

**DOI:** 10.1186/1472-6963-13-102

**Published:** 2013-03-18

**Authors:** Rènée du Toit, Hannah B Faal, Daniel Etya’ale, Boateng Wiafe, Ingrid Mason, Ronnie Graham, Simon Bush, Wanjiku Mathenge, Paul Courtright

**Affiliations:** 1 Eye health consultant, 17 Pecan Place, 831 Mortimer Avenue, Pretoria, Mayville 0084, South Africa; 2 Eye health and health systems consultant, Calabar, Nigeria; 3 MSc Community Eye, International Agency for the Prevention of Blindness, Cape Town, South Africa; 4 MSc (Community Eye Health), Operation Eyesight, Accra, Ghana; 5 MSc (Medical Education), RGN/KRN, CBM, Nairobi, Kenya; 6 DipEd, Sightsavers, Dar Es Salam, Tanzania; 7 MSc(Development Management), Sightsavers, Accra, Ghana; 8 Rwanda International Institute of Ophthalmology, Kigali, Rwanda; 9 The Fred Hollows Foundation, Sydney, Australia; 10 KCCO International, Cape Town, South Africa

**Keywords:** Quality care, Sub-Saharan Africa, Health systems strengthening, Integration, Primary health care, Public health, Eye health, Vision impairment and blindness, Primary eye care, Developing countries

## Abstract

**Background:**

The impact of unmet eye care needs in sub-Saharan Africa is compounded by barriers to accessing eye care, limited engagement with communities, a shortage of appropriately skilled health personnel, and inadequate support from health systems. The renewed focus on primary health care has led to support for greater integration of eye health into national health systems. The aim of this paper is to demonstrate available evidence of integration of eye health into primary health care in sub-Saharan Africa from a health systems strengthening perspective.

**Methods:**

A scoping review method was used to gather and assess information from published literature, reviews, WHO policy documents and examples of eye and health care interventions in sub-Saharan Africa. Findings were compiled using a health systems strengthening framework.

**Results:**

Limited information is available about eye health from a health systems strengthening approach. Particular components of the health systems framework lacking evidence are service delivery, equipment and supplies, financing, leadership and governance. There is some information to support interventions to strengthen human resources at all levels, partnerships and community participation; but little evidence showing their successful application to improve quality of care and access to comprehensive eye health services at the primary health level, and referral to other levels for specialist eye care.

**Conclusion:**

Evidence of integration of eye health into primary health care is currently weak, particularly when applying a health systems framework. A realignment of eye health in the primary health care agenda will require context specific planning and a holistic approach, with careful attention to each of the health system components and to the public health system as a whole. Documentation and evaluation of existing projects are required, as are pilot projects of systematic approaches to interventions and application of best practices. Multi-national research may provide guidance about how to scale up eye health interventions that are integrated into primary health systems.

## Background

Globally about 285 million people are vision impaired [1]. Up to 80% are vision impaired due to treatable or preventable causes [2,3]. Over 90% live in low and middle income countries, and proportionately more in Africa [4]. While the main causes of avoidable blindness and visual impairment may be similar, there is considerable variation in eye care needs, services and numbers and cadres of eye care personnel [5,6] available across Africa, and even in regions within countries [7,8]. In many places there are few health personnel with appropriate competencies; productivity is low, and distribution of resources uneven. In general, the most remote and poorest areas of low-income countries have least access to eye care [3,6,9-16].

In sub-Saharan Africa, health care is available through the public health service. It consists of primary, secondary and tertiary levels, which in most instances focus on providing curative care. The role of the private sector is increasing, mainly in urban settings [17]. Many informal private providers however provide services and medication across Africa [18]. Traditional medicine is widely used in many settings [16,19-26]. Primary health care however proposed an approach that enabled a full range of health care, with prevention equally important as cure, from households to hospitals [27]. After 30 years, the importance of this approach is emerging again [28,29].

Generally only tertiary and some secondary services have specialist eye care services and equipment required to reliably diagnose and manage the major causes of vision impairment [30]. These conditions, which include cataract, refractive error, diabetic retinopathy and glaucoma, usually have a gradual onset. People may not experience or notice symptoms. Alternatively they may use traditional medicine, [16,19-26] self-medicate [18] or develop coping strategies. This may delay presentation to eye care services [31], leading to complications and even irreversible visual loss. A person with these conditions could benefit from earlier identification, counseling and referral [11].

Delays in presentation of other sight threatening conditions, such as injuries, are often due to lack of finances and or ignorance at the community level that interventions are available. This can be compounded by the poor knowledge within the health care sector of appropriate management and the availability of specialist eye care services [16]. Questions about access are also relevant to other commonly occurring ocular conditions, for example allergic conjunctivitis and presbyopia. These conditions do not have sequelae likely to be sight threatening, yet they affect quality of life [32,33]. These issues generate questions about how to facilitate equitable access to eye care at the most appropriate levels [13,16,34].

The WHO considered integration as a key element of primary health care in 1978; [27] Integration remains a cornerstone of initiatives to revitalize primary health care [29]. The WHO defined it as “The management and delivery of health services so that clients receive a continuum of preventive and curative services, according to their needs over time and across different levels of the health system.” There is however no consensus in the peer-reviewed literature on a common definition of integration [35-37]. This may be one of the reasons contributing to the dearth of evidence about the effectiveness of this approach [35,38].

In 1984, the World Health Organization (WHO) recommended a primary health care approach to address issues of access to eye care. This included appropriate management of eye conditions at the primary care level with cascading levels of referral for more complex conditions [39]. From 1999 the VISION 2020 Initiative [40,41], has become the dominant framework guiding eye care [42]. VISION 2020 focuses on priority blinding conditions with the goal of the elimination of avoidable blindness and visual impairment by the year 2020. Primary eye care as an integral part of primary health care was recommended as a key strategy that included “promotion of eye health and/or the provision of basic preventive and curative treatment for common eye disorders”. The role of a primary eye care provider was outlined as the Identification of those blind and vision impaired; assessment and diagnosis for referral; advice about referral and encouragement to attend; follow up: help with rehabilitation, “give advice on any treatment and make sure spectacles are available” [40]. Considerable variance exists in what constitutes “assessment and diagnosis for referral” and “appropriate management of eye conditions at a primary care level”. This may be one of the reasons for the concerns in matching expectations of eye care provision at the primary level with the skills and capacities of providers [14,43,44]. The concept of integration of eye health into primary health care thus enjoys an enabling policy environment, but there is little information about the implementation of these policies.

The World Health Assembly noted in 2009 that significant progress had been made: vision loss due to Vitamin A deficiency [45], trachoma [46] and onchoceriasis [47] had decreased. A review in 2010 however found little published evidence of successful models of primary eye care [30]. A review, thirteen years earlier, [48] had reported only anecdotal evidence of a few small well supported “mission-based” programs that seemed to be more successful than large “government supported” programs.

The call for a revitalized primary health care system, [28] later including eye health in primary health care [49] has been challenged by the often fragile, fragmented and under-resourced systems [50]. The viability of the primary health care systems varies between countries and even between different areas in a country [51]. It has been recommended that health systems should be strengthened to enable most interventions to be delivered in an integrated way, where feasible [37]. Many countries have thus adopted policies using priority health interventions as an entry point to strengthen health systems (health systems strengthening: HSS), based on a primary health care approach [52-54]. The importance of a health systems strengthening approach has been recognized in the eye health literature [55-57].

Although there is broad consensus about its importance, there is also no common definition of health systems strengthening [58]. The WHO defines it by six building blocks that make up the health system but recognizes that these are interdependent [53]. A complexity perspective is thus used to view the interconnectedness and continuous interaction of the components of health systems, and the non-linear effects of the system’s dynamic adjustment [59-61].

**Figure 1:**
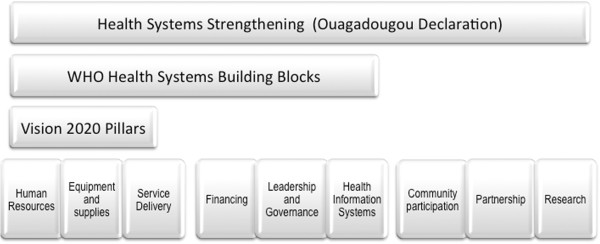
**Overlap between Vision 2020 pillars, health systems building blocks and Ouagadougou key areas.** Areas of overlap between the three pillars of the Vision 2020 initiative, [41,62] and the six WHO health system building blocks, [53] and the nine key areas of Ouagadougou Declaration [63]. See online Additional file 1.

Figure 1 shows the relationship of eye health to health systems and HSS strategies. It depicts areas of overlap between the three pillars of the Vision 2020 initiative, [62] the six WHO health systems building blocks, [53] and the nine key areas of the framework for implementation of the WHO Ouagadougou Declaration on primary health care and health systems [63]. Additional file 1 provides details of, and shows the overlaps between, the three VISION 2020 pillars, the six WHO Health systems building blocks, and the nine elements of the WHO primary heath care approach for strengthening health systems in the African Region (Ouagadougou Declaration on Primary Health Care and Health Systems).

The WHO recommended that international experience be reviewed, lessons learnt and best practices in implementing policies, plans and programs be shared [49]. This paper uses a HSS framework and attempts to describe the scope and breadth of information and evidence available about how eye health has been implemented in primary health care systems.

## Methods

Eye heath interventions occur within complex health systems and are largely context dependent [64]. We therefore used a scoping review because this method provides an opportunity to survey the whole profile of information available for this topic. Further a scoping review method can provide greater clarity about an area such as this, where limited evidence exists, and identify gaps in the evidence [65,66]. The use of a scoping review is new to eye care, but has been used to address questions related to health systems [67,68]. Although scoping reviews on this large scale have a limited utility value for planners / stakeholders at a country level, this paper aimed to identify any existing evidence for pragmatic guidance for planners and policy makers on the implementation of eye health interventions within a primary health care system [65,68].

Scoping reviews, though broad in nature, are intended to guide more focused lines of investigation [65]. Information gathering and analysis was thus theory based and guided using a HSS perspective and WHO frameworks that underlie much of the policy in Africa [53,62,63,69,70].

The scoping review [66] included identifying the research question, identifying relevant studies, study selection, and collating, summarizing and reporting the results. The multidimensional approach that characterizes a scoping review was used to collect different types of information from multiple sources [65,66]. Structured searches of PubMed, the Cochrane library, Health Systems Evidence (McMaster Health Forum) and the WHO site were supplemented by gathering data iteratively, using more informal approaches such as ‘snowballing’ [71]. The review covered 1983 to February 2013.

This paper delineates the provision of eye health as part of the primary health system as:

• occurring from home through to the community and the frontline health facility;

• provided by the health workforce who would include home carers, family members, community health workers, front line health facility based workers;

• in the public/ private, traditional/formal/informal sector;

• and consisting of mainly promotive and preventive services, and of some curative services within a clearly defined scope of practice.

We attempted to identify relevant theory and information regardless of study design, and included both quantitative and qualitative data to obtain a broad overview, but with sufficient depth if possible to facilitate policy lessons. Scoping studies do not discriminate between studies based on methodological criteria [66]. Systematic reviews were however prioritised for inclusion, especially where these contained information from low- and middle-income countries, as were articles pertaining to eye health in Africa.

An iterative process followed to synthesise the information: emerging priorities in eye health in Africa were identified from published information. This was augmented information from review articles and with anecdotes from the authors’ experiences in various African countries. The articles were categorized into the nine priority areas of the framework recommended for the implementation of HSS and primary health care in Africa: human resources for health, health technologies and equipment and supplies, health services delivery, health financing, leadership and governance for health, partnerships for health development, community ownership and participation, health information systems, and research for health [63].

The results are presented as per the HSS framework categories, [52] as a synthesis from the iterative process of analysis.

## Results

None of the papers identified described eye health interventions that were largely provided by routine primary health care systems, or described as an entry point to strengthen health systems in Africa. Four papers described the importance of health systems strengthening with regards to eye health [55-57,72,73]. Table 1 shows that most of the 173 papers included in this review, 43 of which are reviews, contain information from sub-Saharan Africa (76) or from low- and middle-income countries (47).

**Table 1:** Number of papers related to eye health, health systems and integration and reviews

	**Sub-Saharan Africa**	**Low- and middle- income countries**	**High-income country or not specified**
Eye health related	65	8	8
Systematic review		22	10
Review	2	9	
Not eye health related	9	8	6
Health systems			18
Integration			8

### Human resources

Task sharing can contribute to strengthening the health system, by enabling general health care personnel, if they have the appropriate skills, to provide eye care as part of primary health care [74-79]. If too much is added to their routine practice, they may not be able to complete additional tasks [80].

Task-sharing can also extend to lay personnel [81] such as traditional healers, [23,82] school teachers, [83] or community members who are willing to learn additional skills and undertake eye care activities [51,84]. A systematic review found lay health worker care to be associated with promising benefits for maternal and child health and the management of some infectious diseases [85].

Competent and equipped specialist eye care personnel are essential to receive referrals from the primary level. Task sharing with physicians has been used to increase the number of specialist mid-level or allied eye care personnel, such as ophthalmic nurses, and ophthalmic clinical officers [5,6,8,86-93]. Recent reviews used evidence mainly from maternal health and HIV, to show that task sharing to mid-level cadres is a promising strategy and good health outcomes are possible [88,94,95].

The role of specialist mid-level eye care personnel often includes “to train and supervise” other cadres, [49,96] but this review did not find any related information. Information from other fields of health show that few mid-level cadres have either teaching or supervision skills; this supervisory role is rarely fulfilled [97]. Cascade training too has to be well supported to be effective [98].

A study reported a one-day children’s eye health training session for general health staff had encouraging short-term outcomes [99]. In general however, documented longer-term outcomes for eye health training have been less encouraging [14,30,74-76,100]. Evidence from evaluations of the quality of these interventions is sparse: for example whether the skills and knowledge presented in the initial training are appropriate to the context to which health personnel return, or are indeed acquired or retained.

Poor knowledge and skills are broadly attributed to inadequate or inappropriate training, inadequate supervision, or lack of support to implement the eye health skills learned [30,44,100-103]. Trichiasis surgeons in Ethiopia, included refresher training and supervision in their suggestions to increase their output [84]. There is however little evidence as to what the most effective interventions would entail. For example, which of continuing education, equipment, guidelines/ protocols and/or supervision would enable health care providers to provide quality eye care [91]. For obvious reasons, the quality of the intervention is also critical: for example information from both eye health and general health fields suggests providing supportive, problem solving supervision rather than it being a checklist driven exercise [75,76,104]. A systematic review however found insufficient evidence to determine whether managerial supervision has a substantive, positive effect on the quality of primary health care in low- and middle-income countries [105]. Key points provides a summary of human resources’ contribution to primary eye health.

#### Key points: human resources

To develop a functional and sustainable process:

• A functional referral pathway to accessible specialist eye care services is essential.

• Mid-level cadres with adequate equipment and supplies may be best placed to receive referrals, especially if they practice in rural and remote areas.

• If mid-level cadres are expected to have a training and supervisory role, they need training as trainers, resources and support.

• Realistic expectations about which eye care services can most appropriately be implemented and learnt by different cadres in the limited time available during either in-service training or as part of a pre-service course [44,48,106].

• Effective training and assessment to enable individuals to develop competencies appropriate to the context to which they are returning.

• Enabling environment and ongoing support for example for their continuing education, quality improvement, equipment, guidelines/ protocols promotion of teamwork and supportive supervision to enable them to provide quality care [107,108].

### Health technology, equipment and supplies

In addition to appropriate competencies, health personnel who provide eye care require appropriate infrastructure, equipment, equipment maintenance, supplies, [100] an effective supply chain [84] and technology to be able to provide quality care [6,49,100]. Basic equipment and supplies are often lacking; none of the 36 dispensaries surveyed in Tanzania had a visual acuity chart or torch for examination [44]. Lack of equipment has also been identified as a major influence on the productivity of cataract surgeons [6]. In Ethiopia, it was intended that after training, health extension workers would integrate trichiasis surgery into routine activities at static-site facilities. This training has been accompanied by significant ongoing financial investment for instruments and consumables; however, only about 3% of surgeons had all the essential items to perform trichiasis surgery [84]. Few resource challenged countries have added eye medications to their essential drug package [RG]. The searches conducted by this paper did not identify any information on procurement, management and maintenance systems.

### Service delivery

Primary level public health activities such as vitamin A distribution, measles immunisation, ivermectin distribution, facial and environmental hygiene [109-111] are part of the development agenda. These can and do make significant contributions to the eye health of the population [45-47]. If the quality of any health service is poor, however, people may not utilise these [112]. Self-medication using pharmacy or traditional remedies [16,19-22] and /or inappropriate management of eye conditions at front line facilities [15,16] may cause a delay in treatment, exacerbate conditions and even cause blindness. Referral pathways too must be in place, effective and, functional [16,30].

Prevention and promotion of eye health are considered important components of primary care [40]. Patient self-management is required for emerging eye health priorities such as diabetic eye disease and glaucoma [2,113-116]. Appropriate advice [15] and health education, effectively provided, may reduce the spread of infectious diseases, prevent injuries and promote eye health [109-111]. There is however little evidence about the provision, efficacy, or impact of eye health promotion activities [114-116]. Evidence from low and medium income countries is limited in general, for example a systematic review could only identify low quality evidence to indicate that home visits and health education may improve immunization coverage [117].

Trachoma elimination provides an example of the scant information available about health promotion and its potential impact. It also illustrates the comprehensive approach that is required rather than reducing health promotion to the isolated provision of eye health education messages. A systematic review included only one study that showed that health education was effective in reducing the incidence of trachoma at six months. Longer-term outcomes were not provided. The scope of the health education intervention was however extensive: it targeted women and school children and included community participation and information, supported by posters and booklets, on personal hygiene, household sanitation, trachoma and its complications, elements of primary health care; and was repeated one week per month for six-months [109].

Health education programs however often increase knowledge, but behaviour change does not necessarily follow [111]. The lack of environmental support was identified as a reason for the lack of implementation of messages about trachoma in a Tanzanian school curriculum [118]. Further a systematic review did not find evidence of the effectiveness of a change in behaviour, i.e. face washing, to reduce trachoma, when not combined with antibiotic treatment [110]. In addition, a decline in trachoma has been shown without any trachoma-specific interventions, however education, access to health care, water and sanitation had improved in a village in the Gambia [119].

In addition, the concept of health education should extend beyond the domain of the health service. When the action required or the behaviour change required is beyond health, there is a need to rethink the concept and see it as community /society development and how health can or should make it everyone’s business [HF].

In most settings in Africa, eye care services are rarely solely horizontal (delivered through the routine health services) or vertical (delivered through largely free-standing programs): horizontal and vertical approaches are combined to create diagonal services (intervention priorities are used to initiate required improvements to the health system). These may support the development of integrated health systems [11,37,42,120-126]. In this way the routine services at primary and community level are sometimes augmented with specialist eye health outreach visits to more remote settings [30,127]. In general, outreach services have been associated with improved access, health outcomes, more efficient and guideline-consistent care, in particular when delivered as part of a multifaceted intervention that includes other services and education [123,128]. There are some examples however, of eye health outreach services undermining local services and not always providing access to more vulnerable populations [127,129]. If vertical programs are at odds with national health policy, this may limit scalability and there may be consequences due to the resource intensive nature of these interventions [75,130].

There is very little evidence to guide decisions about the most effective delivery strategy: [84] how vertical programs affect horizontal efforts in strengthening health systems or how these can support each other effectively and efficiently [84,125,131] or be combined into diagonal services [11,42,120-126]. A systematic review found some evidence in low and middle-income countries that utilisation and outputs of healthcare delivery may improve when a service is added to an existing service. No evidence was available however to show that healthcare delivery or health outcomes are improved by a full integration of primary health care services. This has been attributed to the decrease in the knowledge and utilisation of specific services that may accompany integration [38]. There is however insufficient evidence to show improvements in outcomes in patients with multi-morbidity in primary care and community settings [132]. The eye health program in Pakistan reported some challenges in aligning eye health with the national health systems, but attributes the success of their program to their health systems strengthening approach and integration into primary health care [73].

### Health financing

In 2001, African governments committed to increase investments in health to 15% of national budgets by 2015; by 2011, only six had achieved this target. A systematic review of low- to middle-income countries calls for more rigorous research to confirm the increase in the utilisation of healthcare services with the removal or reduction of user fees, and vice versa; the unintended consequences such as utilisation of preventive services and service quality; also the beneficial effects of introducing or increasing fees together with quality improvements [133].

Ghana established a National Health Insurance Scheme in 2003 based on district-wide mutual health insurance schemes. These now operate across all districts in the country. The NHIS reported that by the end of 2008, 61% of the population was covered [134]. The indigent and those living in rural areas are however least likely to subscribe [135]. Over 95% of the most common disease conditions are included in the benefits package. Eye care benefits include cataract and eyelid surgery, biometry, visual fields, refraction, and basic ophthalmic preparations, but not optical devices. Implementation of the NHIS has increased access to public health care services and raised public expectations: enrolled individuals are more likely to seek care for illness or injury. Funds for public health activities are however not being increased [136].

Despite the National Health Insurance Scheme, eye care service provision at the district and sub district level is often not optimal. Ophthalmic nurses are generally poorly equipped, and sometimes the only eye health provider in an entire district. It has been reported that these personnel may be discouraged by a lack of supervisory visits, having to cope with the increased workload, without any additional compensation while they perceive the institution’s revenue to be increasing. Facilities often do not stock ophthalmic medications because of their low turnover, necessitating that patients purchase their medication [BW].

In Rwanda, eye health indicators have remained largely static despite more than 400 Health Centre nurses, having attended eye health training over the past two years. Eye care indicators are not included in the performance based incentives scheme. Nurses may thus prioritize activities linked to incentives and over eye health. The Ministry of Health supports a proposal to include eye health indicators in the list of performance based incentives services (e.g., successful detection and referral of those with cataract). The Ministry has requested funding from NGOs for the performance based incentives “basket” to support the “sale” of the eye health indicators [WM]. A systematic review however concluded that more evidence is needed from low- and middle-income countries about both the outcomes and unintended consequences of paying for performance [137] and about the effect of adequate remuneration and non-financial incentives such as recognition and support by regulatory bodies / health systems [88,138].

### Leadership and governance

There is very little information about leadership and governance in primary eye care — in policy setting or in implementation to ensure quality care [139]. In low and middle-income countries, a high burden of eye injury is associated with a lack of safety regulation, lack of awareness of prevention, and potentially harmful social norms of the community [114]. Furthermore, in eye care, as in other fields, service quantity and coverage have at times been prioritised over quality [140]. Quality care is however required to best utilise limited resources [141].

Clinical governance has been described as “changing the way people work; demonstrating that leadership, teamwork and communication is as important to high quality care as risk management and clinical effectiveness” [142]. It includes professionalism and accountability, which may not always be apparent, for example reflected in the high rate of absenteeism among a sample of primary health care workers in Tanzania [44].

### Public private partnerships and multi-sectoral collaboration

The nearly 30 year global and public-private/corporate partnership for mass drug administration for onchocerciasis has been effective [143,144] in reducing the burden of this disease. More recent partnerships for trachoma control, which includes integration of mass drug administration, surgery for trichiasis and hygiene, water and sanitation initiatives have also led to a reduction in the global burden of disease [145]. Both demonstrate the link between eye care and the basic health needs and objectives of the Millennium Development Goals [146]. The World Bank ascribes the success of the African Program for Onchocerciasis Control (APOC) in controlling onchocerciasis to both “the partnership approach to organization, in which countries, civil society, the private sector, donors and UN agencies all play key roles, and the community approach to implementation, which places the program in the hands of its beneficiaries” [147].

Collaboration between eye health and other organisations or health departments such as environmental services, health promotion, nutrition, and social science may facilitate more effective use of resources. Multi-sectoral collaboration, with an emphasis on prevention has been shown to effectively address other eye conditions such as diabetes and many causes of childhood blindness [42,148].

A further source of collaboration has been with traditional healers, [19,23-26,149] however, not all ophthalmologists favour this approach [82]. Nevertheless, it has been shown to be effective: in Malawi, in the year after the collaboration with traditional healers, there was an 80% increase in cataract blind patients presenting to the hospital [25]. Key informants, who may be members of the community, can identify blind children [80,150]. This paper did not find any evidence of eye health collaboration with informal private providers to improve access to quality care [18].

### Community participation

The credibility of a community health worker approach has been damaged due to many programs that have failed unnecessarily. Factors contributing to failure include a lack of understanding of community work, unrealistic expectations, poor planning and underestimation of the finances, resources, and support required for a successful program [97,151-155]. Community consultation has been shown to be important: if an intervention did not address an unmet need of the community, it was less likely to be successful [156].

With changes in society, consumer awareness, and expectations of quality eye health care throughout Africa community consultation has had to be dynamic [30,157,158]. Community health workers drawn from the community and integrated in the primary health care system of the area, contributed to the significant reduction in active trachoma in the north of South Africa the 1980s [77,159]. Below provides an example of a successful community based approach to eradicate onchocerciasis, a condition affecting communities beyond the reach of conventional health systems. The 77% reduction in the prevalence of onchocerciasis is reportedly due to a network of almost 500 000 community volunteers, being able to reach nearly 60 million people across 16 endemic countries in Africa [147].

#### Example of a successful community based intervention: the Onchocerciasis program

The community based and directed interventions, based on distribution of drugs to endemic communities, is a demonstration of the role of the community as stewards, providers and consumers of health services. The host of enabling factors that made this program successful include:

– the recognition of the power of and a respect for communities.

– coordination of activities and strengthening of links with other health services.

– solid program design including realistic expectations, careful selection, appropriate training, monitoring, evaluation and supportive supervision [115,160].

– focus on increased efficiency: combining drug distribution networks, aligning financial resources, integrated training and involvement of community members [161].

Benefits to the health system have included:

– strengthened leadership, improved financing arrangements and training, and appreciation of community ownership [51].

– health personnel being more engaged in outreach activities [51].

Benefits to the community have included:

– increasing awareness of public health issues, understanding of their right to access services and the availability of a wide range of health interventions at no additional costs.

– increasing active participation of women in community activities [162].

### Health information systems

There is little primary research about eye health information systems [55]. Regular and continuous supervision, coupled with responsive monitoring and evaluation systems should ideally be integrated in national health care systems [39,97,163]. This could provide information to analyse the health system response to various needs (logistic, supplies and training) [97] and for devising quality improvement strategies [164] and identify learning gaps for continuing professional development [165,166]. For example, regular clinical audits to collect information on the clinical outcomes of cataract surgery, and also patient reported outcomes collected with a validated instrument, and using this information to improve services, should be a routine part of cataract surgical services [140].

While there is insufficient evidence to show which strategies promoting the use of information technology are most effective at guiding implementation of health information systems [167-169] a new eye health information system in Kenya provides an example of its application to improve eye related health information collection and use. Until recently, all eye care data in Kenya were collected manually and mailed to the Division of Ophthalmic Services at the Ministry of Health. Even when postage was pre-paid, the response rate remained low: 39% in 2007. Pilot projects, in three eye units in Kenya, provided training for eye health personnel, computers with health information software and internet connectivity via mobile phones, at around $3 a month. Initially eye care personnel were reluctant to use the system because of the perception of additional work required. Reluctance receded when they recognized that they could easily access their data and generate their routine reports. Nine hospitals are now enrolled and the Division of Ophthalmic Services reports that accurate, timely and useful information is now available to generate reports, to facilitate decision-making and to plan expansion of eye services [WM].

### Research

Given the complexities of health care systems and the variable context in which systems are embedded, integration of primary eye care into primary health care is not likely to be simple [130,170]. At the present time, there is insufficient evidence to guide policy makers and planners on how to strengthen health systems, to improve the performance of systems, measure health impact, or to show that health service delivery or health status improves with integration [38]. Furthermore, there is little evidence of existing research findings being used to change policies and practices. A review of systematic reviews to determine how to increase coverage and access to cataract and other health services in developing countries identified the need for quality primary research on health systems [55].

## Discussion

Even though health system strengthening (HSS) is considered an international priority, [171] there is little evidence of its successful application [58,172], or of how ‘everyone’ can contribute [53]. For example it is recommended that health personnel should identify ways to collaborate with peers, advocate for change and engage with the health system [56,57]. There is however insufficient evidence about how health care providers, as active agents of change within a complex health system, can influence health systems. There is some indication that strategies that provide support for quality and performance improvement and change the accountability of individual informal private providers in low- and middle-income countries, are more likely to be successful than those that depend on training [18].

Furthermore, evidence is lacking about the delivery of comprehensive eye health services or of the effectiveness of eye care service provision within either vertical or horizontal approaches [14,30,58,74-76,99,100] or the procurement, management and maintenance systems for equipment and supplies. This may be due to a lack of documentation, sufficient research, application of available research for planning [173], or to the genuine lack of effective eye care interventions at primary level.

There is some evidence however about what does not work, particularly regarding human resources, service delivery, and equipment. This appears to be related to a wide array of factors including inadequate content and quality of training and support, the inability of lesser trained and equipped eye health providers to detect and refer problems accurately or sufficiently early and difficulty in restricting primary health care providers to appropriately manage only simple, uncomplicated cases [30,49]. For example improving and /or adding eye health training into the training of primary health care providers, may thus be insufficient and may even have negative consequences if this is perceived as an ‘extra’ duty. Eye health has to be perceived as part of overall health. Further concurrent strengthening of the overall primary health care system of supervision and support and of the other blocks of the health system is required [HF].

The weaknesses within the eye health system blocks and lack of evidence of how to most effectively strengthen these blocks, thus emerge as challenges to making recommendations about how these can best contribute to HSS and integration. Information that is available, for example from Community Directed Interventions for onchocerciasis [124,162,174,175], is invaluable. The health system, however does not function as isolated blocks, these are interdependent and interact with each other. Strengthening only a single block is thus unlikely to be successful. A holistic approach to HSS is required and all the components need to be addressed to maximize outcomes [70]. The sustainability of eye care gains will thus depend on how eye health can contribute to the strengthening of the overall structure and performance of the national health system [HF].

### Strengths and limitations of this review

Most of the published work about eye health originates from eastern and southern Africa and there is a pronounced lack of information from central and western Africa. Although this review found many articles about eye health in Africa, few specifically addressed health systems and integration. Many of the systematic reviews contained information from higher-income countries and from fields such as maternal and child health and HIV, rather than eye health. Many health systems challenges to the provision of quality eye care in sub-Saharan Africa are similar to those faced by other parts of health care, in many other low and medium income countries. These include providing adequate resources and support, equitable access to eye care services and sufficient appropriately skilled health personnel [2,4,13,33,34,176-184]. Community Directed Interventions for onchocerciasis [124,162,174,175], and trachoma programs have overcome many of these challenges and we can learn from their strengths, the extent to which these work and how and in the circumstances in which these work [185,186]. Also from evidence from other countries and other fields: for example how nurses in high-income countries provide safe and effective eye care [187-193].

The information gathering and analysis process for this paper was guided by a scoping review method using a HSS framework [66]. Searches of three databases were undertaken in an attempt at comprehensiveness as well as consultation with an expert group, the focus being on examples of integration of eye health into primary health care. This review included a broad range of study designs, systematic reviews and also relevant theoretical and qualitative work. In addition, integration of information and reporting was theory based to enhance it utility [185]. This enabled us to capitalize on the strengths of a scoping review method: “to extract the essence of a diverse body of evidence and give meaning and significance to a topic that is both developmental and intellectually creative” (p1398) [65]. Consequently, the information can assist the eye care community and policy makers to consider the complexities and challenges of integration of eye health into primary health care in Africa and the contribution eye health can make to HSS.

One of the limitations of scoping reviews is that these do not formally assess the quality of evidence; consequently, it is not possible to determine the robustness or generalisability of the findings. Also ‘synthesis’, i.e. the relative weight of evidence in favour of the effectiveness of a particular intervention is not addressed. Further these provide a narrative or descriptive account of available information, thus are open to different interpretations by readers [65,66]. There is however limited information to guide the review of complex interventions: about the best approach to the synthesis of data and issues such as the standardization of study selection, and techniques for the quality assessment of less conventional study designs [185].

This paper had a very broad remit – dictated by the holistic approach that is proposed by the HSS perspective. By virtue of the information available, it concentrated on particular aspects of each of the HSS components; a detailed account of context and process of all implementations were necessarily curtailed by the breadth of the topic. Also the linkages and interrelated nature of the components, and the complexity of primary health care as a multi-dimensional system, were less apparent [70,170]. That said, this review gathered together evidence from a variety of sources. These may otherwise have remained dispersed. The findings indicated the scope to focus this search to uncover further evidence about specific topics and to discover what works and where. In addition it also identified the additional research required to assist policy makers and planners in determining how best to integrate eye care into primary health care systems.

## Conclusion

Given the vast differences in context [58,170,186] between countries in sub-Saharan Africa and between regions in any country, any generic recommendations should be made with caution. There is however sufficient information to indicate that it is unreasonable to expect that quality eye care could be available at the primary level, after only a single episode of perfunctory training. This is particularly true when this training occurs with little consideration of the state of the health system and support for implementation, such as referral systems, supervision, equipment and supplies and with minimal appreciation of community expectations. These shortcomings, it should be noted, are by no means unique to the eye health component of PHC and can be generalised to overall weaknesses in the health system. Operational research and knowledge translation and advocacy to stakeholders may be required to facilitate the shift away from the concept that a primary eye care approach is simple and all that is required is a single ready-made manual [194].

The reality of fragile health systems in many countries [50] and a lack of evidence about the effectiveness of an integrated approach to primary health care [38] may dictate an incremental approach to initiating or integrating eye health interventions into the primary health care system [195].

Suggestions for areas of further research about best practice for integration of eye care into primary health care systems from a HSS standpoint include:

• Generation of evidence of effectiveness and efficiency from existing eye health projects to identify trends or best practices [49,196].

• Conduct and assess evidence-informed pilot projects. This could include investigations such as the competencies required, the potential of new technology to augment the workforce and meet changing community and eye health needs [168,169,197-200].

• Use of common training and implementation protocols in multi-national settings to identify parameters of success common to all [201,202]. This will help determine evidence-based processes applicable for “scale up” [175,186].

• Multinational research can also provide information on context dependency, such as the robustness of the primary health care system, of complex health and quality improvement interventions [203].

• Testing of the application of a few meaningful indicators; these should measure both process and outcome and include various aspects such responsiveness of the eye care services, utilization of eye health services and quality care [128]. The eye health systems assessment tool includes indicators that can be used to assess a country’s eye health system with the aim of promoting eye health systems strengthening interventions [204].

• Eye health personnel have an active role in the periodic evaluation of both the process and the outcome of integration of eye care from a HSS perspective — to identify gaps and plan improvements to their performance and quality of care [55,56,141,164].

At the present time, there is very little evidence to guide the integration of eye care into the primary health care system, particularly when applying a HSS approach. Evidence that exists is at times contradictory, particularly when considering the HSS components of human resources, equipment and supplies, and service delivery. There is strong evidence of importance of partnerships and the participation of the community that are applicable to the integration of eye care into primary health care. While there is evidence from other fields of health care on the importance of other HSS components (financing, information, leadership and governance and research) there is scant evidence regarding eye care.

A series of regional meetings held between 2006 and 2010 in eastern, western, central and southern Africa enabled representatives from 36 of the 46 countries of Sub-Saharan Africa to share their experiences of primary eye care. During these meetings a definition of primary eye care was crafted and refined by the International Agency of the Prevention of Blindness Primary Eye Care Working Group. This definition has also been endorsed by the WHO’s regional office for Africa.

Primary care for eye health is an integrated, participatory and inclusive approach to the eye health component of primary health care consisting of promotive, preventive, curative and rehabilitative services. It is delivered by the health workforce (formal and informal) in conjunction with community members, up to and including services at the front-line health facilities.

A common definition may promote a shared concept of this complex area, and assist in facilitating communication, shared activities and research. Available information and evidence could provide a roadmap guide to the design of eye health interventions integrated into primary health — to provide access to quality eye care services at the primary level. A realignment of eye health in the primary health care agenda will require a holistic approach, with careful attention to each of the health system components and to the public health system as a whole [70].

## Competing interests

The authors declare that they have no competing interests.

## Authors’ contribution

RduT made substantial contributions to conception and design, acquisition of data, and interpretation of data; drafted the manuscript, and saw and approved the final version. BW, SB, RG, IM, WM contributed to content of the paper and revised it critically for substantial intellectual content and saw and approved the final version. HF, DE, made substantial contributions to conception and contributed to content of the paper and revised it critically for substantial intellectual content and saw and approved the final version. PC made substantial contributions to the design and contributed to content of the paper and revised it critically for substantial intellectual content and saw and approved the final version. All authors read and approved the final manuscript.

## Authors’ information

The authors are members of or contributors to the International Agency for the Prevention of Avoidable Blindness Primary Eye Care Working Group.

## Pre-publication history

The pre-publication history for this paper can be accessed here:

http://www.biomedcentral.com/1472-6963/13/102/prepub

## Supplementary Material

Additional file 1 Primary Health Care and Strengthening Health Systems in Africa. Click here for file
