# Supplementary material for: Evidence for integrating eye health into primary health care in Africa: a health systems strengthening approach
Source: BMC Health Serv Res. 2013 Mar 18;13:102. doi: 10.1186/1472-6963-13-102 (PMC3616885; doi:10.1186/1472-6963-13-102)
Supplement: Additional file 1 — Primary Health Care and Strengthening Health Systems in Africa. [file 1472-6963-13-102-S1.docx]

Additional file 1: Primary Health Care and Strengthening Health Systems in Africa

| **Vision 2020 pillars** | **WHO Health systems building blocks** | **Health systems strengthening (Ouagadougou Declaration on Primary Health Care and Health Systems)** | ***health services delivery***   - Develop models, stakeholder coordination including referral systems service organization to promote integration and strengthened efficiency and equity. - Focus on local health system development to improve access, equity and quality of health services |
| --- | --- | --- | --- |
|  |  |  | ***management of human resources for health (HRH)***   - Elaborate comprehensive evidence-based health workforce planning and monitoring; - Build capacity for health training for scaling up the training of relevant cadres of health care providers; HRH management and leadership; and mobilize resources for HRH development. |
|  |  |  | ***health technologies and equipment***   - Increase access to quality and safe health technologies; elaborate national policies and plans on health technologies; develop norms and standards for selection, use and management of appropriate health technologies - Institute a transparent, sustainable and reliable system for the procurement of health-technologies and for increasing availability, affordability and accessibility of essential medicines, commodities, supplies, infrastructure through provision of adequate resources, technology transfer, South-South cooperation, use of community-directed approaches, African traditional medicines |
|  |  |  | ***leadership and governance for health***   - Institutionalize inter-sectoral action for improving health determinants; - Update comprehensive National Health Policy in line with the Primary Health Care (PHC) approach and other regional strategies; the national health strategic plan to ensure integrated management; and providing comprehensive essential health services. |
|  |  |  | ***health system financing***   - Elaborate comprehensive health financing policies and plans, integrated into the overall national development framework, that protect the poor and vulnerable, in particular women and children, while ensuring equitable and sustainable allocation of resources by level of care and the right balance between promotive, preventive, curative and rehabilitative care - Institutionalize national health accounts and efficiency monitoring; strengthen financial management skills at all levels; implement Paris Declaration on Aid Effectiveness. - Implement the Abuja Declaration to incrementally allocate at least 15% of the overall national budget to health; allocate at least 2% of the health budget to reinforce national health research systems and create centres of excellence in Africa - Develop and implement national health insurance schemes that prevent catastrophic health expenditures and ensure solidarity and social protection; |
|  |  |  | ***national health information systems (NHIS)***,   - Elaborate and implement a comprehensive NHIS policy and plan; improve the availability of competent health statistics staff; leverage population surveys to strengthen information - Integrate health information sub-systems, strengthen linkages between ministries of health and other ministries. - Strengthen health information and surveillance systems and promote operational research on health systems for evidence-based decisions |
|  |  |  | ***community participation*** and *empowerment of communities in health development to improve their well-being;*   - Create an enabling policy framework for community participation; build community capacity; reorient the health service delivery system to improve community access and utilization; and use health promotion strategies to empower communities to adopt healthier lifestyles. - Promote health awareness; build the capacity of communities to change behaviours, adopt healthier lifestyles, take ownership of their health and be more involved in health-related activities; and create an environment to empower communities in the governance of health care |
|  |  |  | ***partnerships*** for health development   - Use mechanisms such as the International Health Partnership Plus and the Harmonization for Health in Africa initiatives to promote harmonization and alignment in line with PHC approach - Adopt inter-sectoral collaboration, public–private partnership and civil society participation in policy formulation and service delivery, including civil society and communities with a view to improving the use of health services and taking appropriate action on the economic, social, demographic, nutritional, cultural and environmental determinants of health including climate change; also reaffirming and recognizing the importance of a concerted partnership, in particular, civil society, private sector and development partners to translate commitments into action |
|  |  |  | ***research for health t***o improve the generation and utilization review structures and mechanisms for implementing research for health and knowledge systems;   - Improve South–South and North–South cooperation; create a critical mass of national health researchers; - Allocate adequate funding to research for health; and translate research results into policy and action |
